# Comparison of Respiratory Health Impacts Associated with Wood and Charcoal Biomass Fuels: A Population-Based Analysis of 475,000 Children from 30 Low- and Middle-Income Countries

**DOI:** 10.3390/ijerph18179305

**Published:** 2021-09-03

**Authors:** Katherine E. Woolley, Suzanne E. Bartington, Telesphore Kabera, Xiang-Qian Lao, Francis D. Pope, Sheila M. Greenfield, Malcolm J. Price, G. Neil Thomas

**Affiliations:** 1 Institute of Applied Health Research, University of Birmingham, Edgbaston, Birmingham B15 2TT, UK; KEW863@student.bham.ac.uk (K.E.W.); S.M.Greenfield@bham.ac.uk (S.M.G.); M.Price.2@bham.ac.uk (M.J.P.); g.n.thomas@bham.ac.uk (G.N.T.); 2 College of Science and Technology, University of Rwanda, Avenue de l’Armee, Kigali P.O. Box 3900, Rwanda; kaberacris@yahoo.fr; 3 The Jockey Club School of Public Health and Primary Care, The Chinese University of Hong Kong, Hong Kong; xqlao@cuhk.edu.hk; 4 School of Geography, Earth and Environmental Sciences, University of Birmingham, Edgbaston, Birmingham B15 2TT, UK; F.Pope@bham.ac.uk; 5 NIHR Birmingham Biomedical Research Centre, University Hospitals Birmingham NHS Foundation Trust and University of Birmingham, Birmingham B15 2TT, UK

**Keywords:** acute respiratory infection, biomass fuel, household air pollution, respiratory symptoms, low-and middle-income countries

## Abstract

Background: The World Health Organisation reported that 45% of global acute respiratory infection (ARI) deaths in children under five years are attributable to household air pollution, which has been recognised to be strongly associated with solid biomass fuel usage in domestic settings. The introduction of legislative restrictions for charcoal production or purchase can result in unintended consequences, such as reversion to more polluting biomass fuels such as wood; which may increase health and environmental harms. However, there remains a paucity of evidence concerning the relative health risks between wood and charcoal. This study compares the risk of respiratory symptoms, ARI, and severe ARI among children aged under five years living in wood and charcoal fuel households across 30 low- and middle-income countries. Methods: Data from children (*N* = 475,089) residing in wood or charcoal cooking households were extracted from multiple population-based Demographic and Health Survey databases (DHS) (*N* = 30 countries). Outcome measures were obtained from a maternal report of respiratory symptoms (cough, shortness of breath and fever) occurring in the two weeks prior to the survey date, generating a composite measure of ARI (cough and shortness of breath) and severe ARI (cough, shortness of breath and fever). Multivariable logistic regression analyses were implemented, with adjustment at individual, household, regional and country level for relevant demographic, social, and health-related confounding factors. Results: Increased odds ratios of fever (AOR: 1.07; 95% CI: 1.02–1.12) were observed among children living in wood cooking households compared to the use of charcoal. However, no association was observed with shortness of breath (AOR: 1.03; 95% CI: 0.96–1.10), cough (AOR: 0.99; 95% CI: 0.95–1.04), ARI (AOR: 1.03; 95% CI: 0.96–1.11) or severe ARI (AOR: 1.07; 95% CI: 0.99–1.17). Within rural areas, only shortness of breath was observed to be associated with wood cooking (AOR: 1.08; 95% CI: 1.01–1.15). However, an increased odds ratio of ARI was observed in Asian (AOR: 1.25; 95% CI: 1.04–1.51) and East African countries (AOR: 1.11; 95% CI: 1.01–1.22) only. Conclusion: Our population-based observational data indicates that in Asia and East Africa there is a greater risk of ARI among children aged under 5 years living in wood compared to charcoal cooking households. These findings have major implications for understanding the existing health impacts of wood-based biomass fuel usage and may be of relevance to settings where charcoal fuel restrictions are under consideration.

## 1. Introduction

Exposure to household air pollution (HAP) is associated with adverse child and maternal health outcomes, including morbidity and mortality in children under five years old [1,2,3], acute respiratory infection (ARI) [4], child growth failure [5], low birth weight, and stillbirths [6]. Vulnerability to ARI, the leading cause of mortality in children under five years worldwide [7], is high among children due to a greater level of pollutant inhalation from the same external concentration as their adult counterparts, and more susceptible pulmonary physiology [4]. HAP includes carbon monoxide (CO), particulate matter (PM), sulphur dioxide (SO_2_) and nitrogen dioxide (NO_2_) produced from burning biomass (wood, dung, charcoal and crop residue) for cooking, heating and lighting. Despite the known high HAP exposures in low- and middle-income countries (LMICs), there are few sustainable “cleaner fuel” interventions available to these populations, due to multiple barriers to sustained uptake, including low financial and infrastructural capabilities, lack of awareness, and appropriate policies [8]. Research has indicated harm reduction approaches such as outdoor cooking [9,10,11,12] and cooking with charcoal compared to wood [13,14], lowers exposure; with two small scale studies rural/peri-urban and urban settings, providing evidence for a respiratory health difference between wood and charcoal users [15,16]. However, the evidence in support of such approaches in LMIC settings remains limited.

Some governments have adopted legislative approaches to restrict the use of charcoal due to the recognised environmental and health impacts [17]. Evidence from domestic and commercial kitchens suggests that charcoal cooking is associated with high levels of PM [18] and CO [19] above the World Health Organisation’s Indoor Air Quality Guidelines (WHO-IAQ) [18,20]. Introduction of charcoal fuel-based legislative changes or fiscal disincentives are typically intended to improve population health, including shifting to cleaner fuels such as electricity and liquid petroleum gas (LPG) alternatives. However, such changes may also generate unintended consequences [21,22], such as substitution with more polluting biomass fuels (e.g., wood, dung, straw) [23] which are typically readily available and cheaper alternatives [24]. Wood is the most common fuel used globally and it is therefore preferred as it suits traditional cooking practices [25]. In addition, LPG adoption is not likely in the imminent future due to multiple barriers, including equipment and fuels access, cost, and safety concerns [26]. Societal and economic issues with uncertainty can also affect fuel choices [27,28], meaning fuel transition often does not occur in a linear fashion [29,30]. In the advent of policy measures to restrict charcoal use, it is possible that charcoal could be replaced by wood fuel by end-users, presenting overall health risks given that wood produces more PM than charcoal [18]. But there remains a paucity of evidence in the relative health effects between wood and charcoal cooking, on a global scale.

We report the association of under-five respiratory health (respiratory symptoms, ARI, severe ARI) with wood and charcoal fuel use for cooking, in over 475,000 children from 30 LMICs, using comprehensive population-based data obtained from the Demographic and Health Survey (DHS).

## 2. Materials and Methods

### 2.1. Data Sources

**Figure A1:**
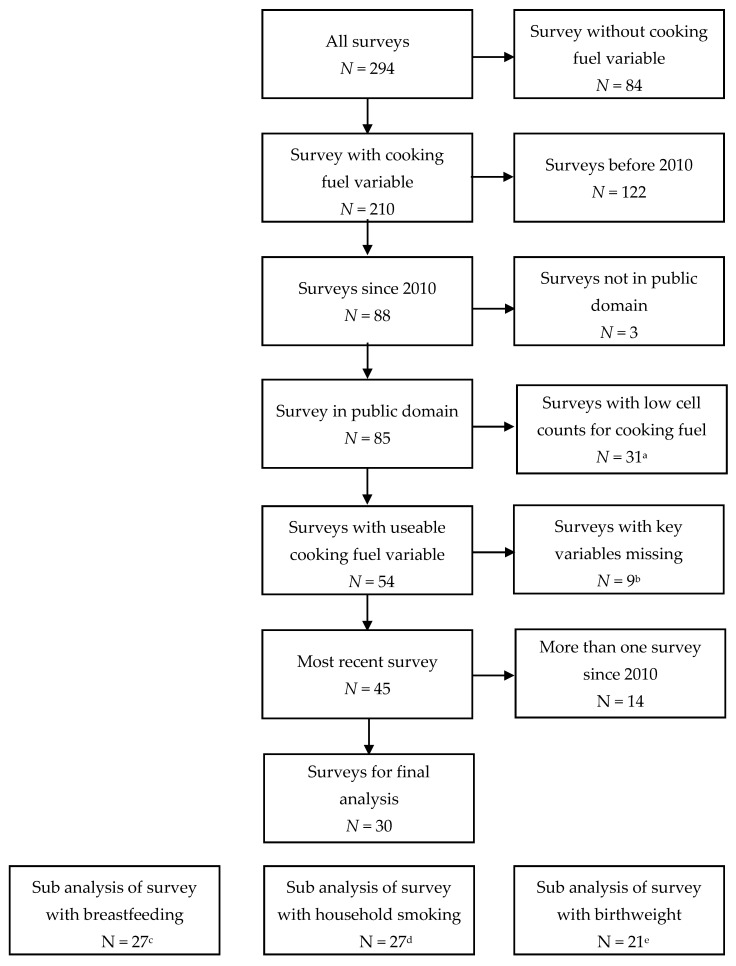
Flow diagram for selection of final dataset for analysis from all household data sets available from the DHS program data archive [31] (**a**) Survey was excluded due to have low cell counts for wood or charcoal cooking (*n* = 31) included: Comoros 2012, Zimbabwe 2010–2011, Zimbabwe 2015, Senegal 2010–2017 (continuous dataset), Togo 2013–2014, Kyrgyz 2012, Tajikistan 2012, 2017, Papa New Guinea 2016–2017, Bangladesh 2011, 2014, Indonesia 2017, Maldives 2016–2017, Nepal 2011, 2016, Timor-Leste 2016, Albania 2017–2018, Armenia 2010, 2015–2016, Jordan 2012, Yemen 2012, Columbia 2010, 2015, Guatemala 2014–2015, Honduras 2011–2012, South Africa 2016, Namibia 2013, Lesotho 2014. (**b**) Surveys were excluded due to low cell counts with explanatory variable (*n* = 3) and low cell counts of missing entirely for wealth (*n* = 3) or three or more key variables (e.g., Breastfeeding, mode of delivery, household smoking) (*n* = 3). (**c**) Peru, Zambia and Kenya were excluded due to <50% missing data for breastfeeding. (**d**) Household smoking data was not collected for Kenya, Peru and the Philippines. (**e**) Chad, Ethiopia, Kenya, Guinea, Liberia, Afghanistan, Myanmar, Pakistan, Nigeria were excluded due to <50% missing data for birthweight.

A cross-sectional study across 30 LMIC countries was conducted using data obtained from the most recently available national population-based Demographic and Health Survey (DHS) [31], with LMIC status defined using the Development Assistance Committee (DAC) list 2020 [32]. Criteria for country inclusion included: (i) DHS survey data available from within the last 10 years, (ii) presence of wood and charcoal cooking fuel use (iii) presence of the outcome variables of interest (Appendix A: Figure A1). Each country followed the same two-stage stratified DHS sampling methodology with proportionate random sampling and standardised questionnaires with fieldwork supported by United States Agency for International Development (USAID). Eligible participants were identified through the residential household survey and included ever-married (has been married at least once in their life) women aged 15–49 years and men aged 15–59 years, who resided in the household the night before the survey [33]. Non-response households at the time of data collection and those with institutional living arrangements (e.g., boarding schools, police camps, army barracks, and hospitals) were excluded.

All countries followed the standard core questionnaire from Phases VI, VII, and VIII of the DHS Program model, with country-specific modifications to non-core questions to reflect the population and health issues most relevant to that country. USAID standardises and provides training to government agencies and health authorities to complete surveys, with internal training and supervision of local data collectors and data entry. The questionnaire is translated into the main language(s) for each country and validated on approximately 100–200 households. Data for this current analysis were obtained from (i) household dataset containing situational and household characteristics; (ii) woman’s dataset containing maternal characteristics; (iii) children’s dataset containing health and individual characteristics. All primary data collection has ethical approval from the relevant government authority within each country, with all data being anonymised and aggregated for DHS online data archive [31]. The archive is publicly available and authorisation for data access has been gained for this study.

### 2.2. Modified Wealth Index

The wealth index provided by DHS is calculated through principal component analysis, including cooking fuel as an indicator variable [34], therefore to prevent effect underestimation due to circularity, a modified wealth index was calculated [35] following the DHS provided guide [36] using SPSS [37], to calculate a modified wealth index. The new wealth index included indicator variables for the source of drinking water, house construction material (wall, roof and floor), toilet facility and assets. The assets included vary by country [37] and have been documented in Appendix B: Table A1. The wealth index was then ranked by household to provide tertiles of wealth.

### 2.3. Outcome Variables-Measure of Respiratory Symptoms and Acute Respiratory Infection

Maternal respondents were asked to report the presence of respiratory symptoms (shortness of breath, cough and fever) during the two weeks prior to the survey among all children under the age of five years living in their household. Respiratory symptoms were modelled as binary outcomes (yes, no), included short rapid breaths or difficulty breathing, cough, and fever. These respiratory symptoms were used to form the composite measures for ARI (both shortness of breath and cough [38]), and severe ARI (each of shortness of breath, cough, and fever [39,40,41]). Composite measures for ARI and severe ARI were then modelled as binary (yes, no) outcomes.

### 2.4. Measure of Exposure to HAP

Cooking fuel use was recorded from self-report for each household that undertook cooking activities. Fuels were categorised as “Cleaner fuels” (electricity, LPG, natural gas, biogas) and “Solid biomass fuels and kerosene” (kerosene, coal/lignite, charcoal, wood, straw/shrubs/grass, agricultural crop, animal dung). Wood and charcoal cooking household fuels were extracted and modelled as a binary variable.

### 2.5. Explanatory Variables

Individual child characteristics included child’s age (0–11, 12–23, 24–35, 36–48, 48–59 months), sex (male, female), mode of delivery (caesarean, vaginal), birth order (first, not first born), breastfeeding status (ever, never), vitamin A supplementation in the last 6 months (yes, no), iron supplementation (yes, no). Maternal characteristics included age of mother (15–24, 25–35, 36–49 years), mother’s highest attained educational level (none, primary, secondary/higher). Household characteristics included: number of household members (≤6, >6), household smoking (yes, no), cooking location (indoor, outdoor), and modified wealth index (lowest, low, middle, high, highest) [34]. Situational variables included geographical region of residence and area of residence (rural, urban). All co-variates were modelled as categorical variables.

### 2.6. Missing Data

Data that were identified to be missing at random with less than 50% missing data [42,43] underwent multiple imputations of 50 iterations [44,45], at a country level, using the MICE package [46] in R studio [47].

### 2.7. Data Analysis

Using R studio [47], descriptive statistics were tabulated with the number of cases (*n*), and percentage (%) for categorical outcome variables within the combined dataset. The association between the health outcome variables and exposure to HAP was assessed using a multivariable logistic regression using the Survey package [48] in R to account for the sampling strategy. Adjusted odds ratios (AOR) and 95% confidence intervals (95% CI) for each country were obtained and presented on a forest plot, with a summary result for the combined dataset. Additional exploratory analyses of a subset of countries were undertaken, incorporating breastfeeding, birthweight, and household smoking, which were missing or incomplete in a number of countries. Stratified analyses were undertaken to investigate the association in rural and urban settings, indoor and outdoor cooking status, geographic location and before or after 2014 (mid-time point of included studies), separately.

## 3. Results

### 3.1. Cooking Fuel Usage and Number of Respiratory Outcomes

**Figure 1:**
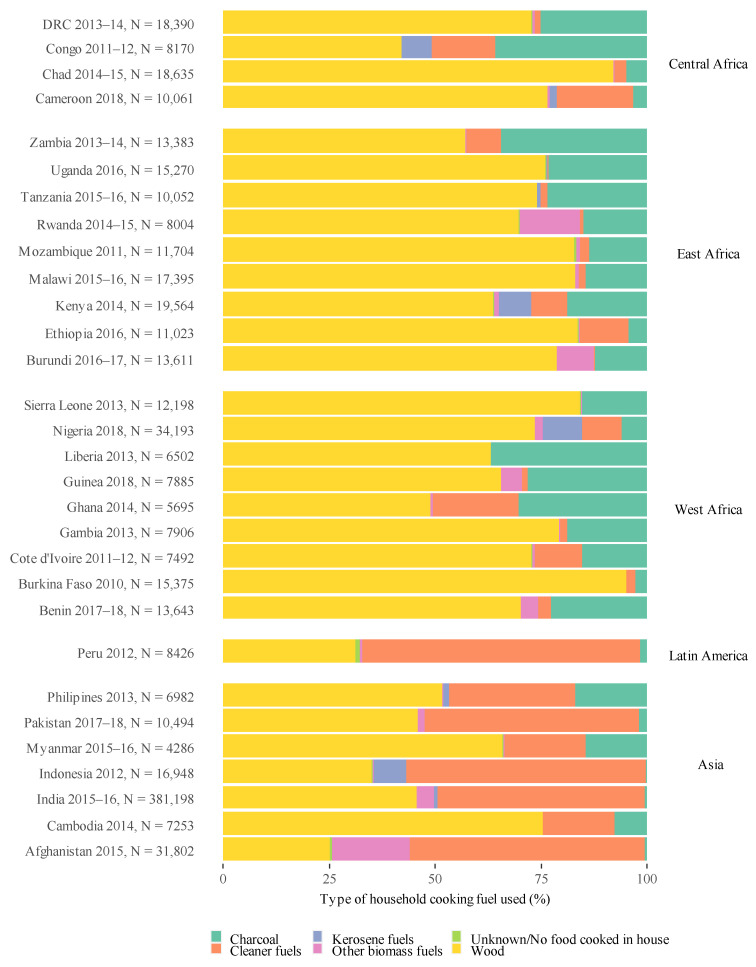
Proportion of clean, kerosene, wood, charcoal, other biomass (dung, crop residue) fuel use within each country, ordered by geographical region.

Out of the 30 included country datasets, there was substantial variation in the type of fuels used within the country (Figure 1), however, wood was the predominant cooking fuel (range: 2.5–94.9%). Indonesia, Afghanistan, Peru, Pakistan and India have a large proportion of “cleaner” fuel use (range: 48.9–56.6%), with low charcoal usage (range: 0.4–2.1%). Within the pooled dataset before imputation (*N* = 475,089), 88.7% used wood cooking fuel compared to 11.1% using charcoal cooking fuel (Table 1). Overall, there were 23,490 cases of severe ARI (5.3%), 36,657 of ARI (8.3%), with shortness of breath being reported in 38,703 children (8.8%), cough in 82,523 children (18.7%), and fever in 89,621 children (20.3%) (Table 1).

### 3.2. Risk of Respiratory Symptoms, ARI, and Severe ARI

**Figure 2:**
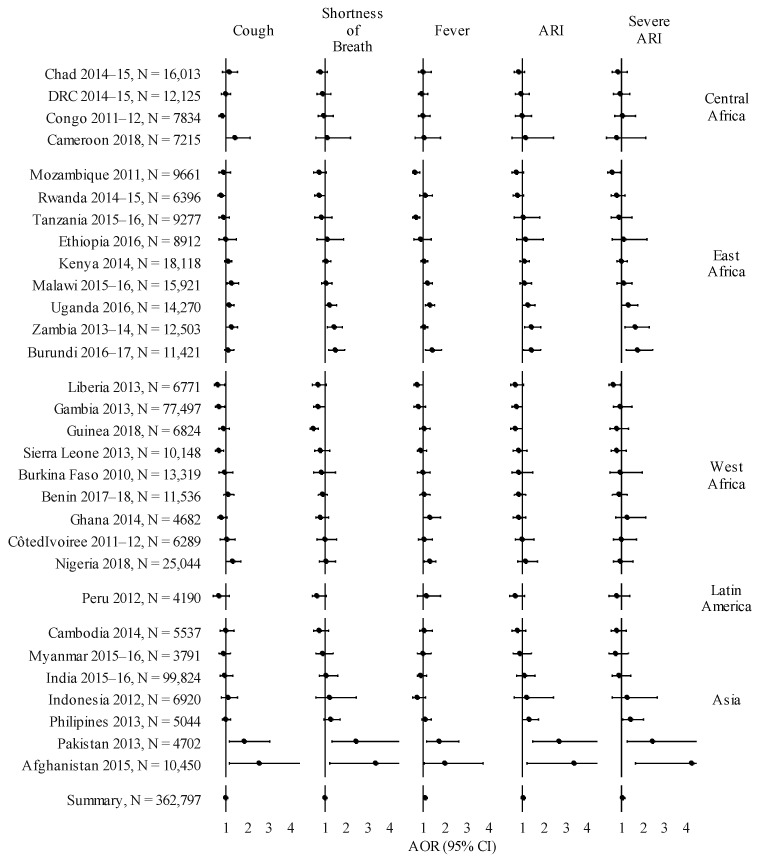
Forest plot illustrating the adjusted odds ratio (AOR) for respiratory symptoms, ARI and severe ARI with wood cooking fuel compared to charcoal for all countries. The summary measure is adjusted for: age, birth order, mode of delivery, vitamin A supplementation, mother’s age, mother’s education level, wealth status, number of household members, rural/urban residence and location of cooking.

After adjusting for individual and situational potential confounding factors, children who resided in wood cooking households were observed to have increased adjusted odds ratios (Figure 2) with fever only (AOR: 1.07; 95% CI: 1.02–1.12), in the pooled dataset. No association was observed with ARI (AOR: 1.03; 95% CI: 0.96–1.11) or severe ARI (AOR: 1.07; 95% CI: 0.99–1.17). However, at a country level Afghanistan (AOR: 4.24; 95% CI: 1.66–10.83), Pakistan (AOR: 2.44; 95% CI: 1.29–4.61), Burundi (AOR: 1.73; 95% CI: 1.21–2.46), Zambia (AOR: 1.62; 95% CI: 1.16–2.26), Philippines (AOR: 1.44; 95% CI: 1.04–2.00) and Uganda (AOR: 1.34; 95% CI: 1.02–1.76), were all observed to have increased adjusted odds ratios of severe ARI in children residing in wood cooking households compared to charcoal cooking. This observed increase in adjusted odds ratios was also present in Afghanistan (AOR: 3.38; 95% CI: 1.23–9.29), Pakistan (AOR: 2.71; 95% CI: 1.45–5.07), Zambia (AOR: 1.43; 95% CI: 1.12–1.83), Burundi (AOR: 1.40; 95% CI: 1.06–1.84) and Uganda (AOR: 1.26; 95% CI: 1.00–1.58) for ARI. Little change was observed in the effect estimate when controlling for birthweight, breastfeeding and household smoking in those countries with available data (Appendix C: Table A2).

### 3.3. Role of Rural and Urban Residence

In the rural and urban sub-analysis cough was observed to be associated with an increased odds ratio (AOR: 1.08; 95% CI: 1.01–1.15) among children residing in wood compared to charcoal fuel households in rural areas only.

### 3.4. Role of Outdoor Cooking

In the analyses of the pooled dataset, for indoor cooking children under five years residing in households using wood had increased adjusted odds ratios of fever (AOR: 1.07; 95% CI: 1.00–1.13). No other differences were observed (Table 2).

### 3.5. Role of Geographic Location

In the stratified sub-analysis by geographic location, an association with ARI in children under five years old living in wood compared to charcoal cooking households was observed in East Africa (AOR: 1.11; 95% CI: 1.01–1.22) and Asia (AOR: 1.25; 95% CI: 1.04–1.51) (Table 2). An increase in the adjusted odds ratio with wood cooking compared to charcoal was also observed with shortness of breath in Asia (AOR: 1.25; 95% CI: 1.04–1.51) and East Africa (AOR: 1.10; 95% CI: 1.00–1.20), whereas a decrease in the adjusted odds ratio was observed in West Africa (AOR: 0.86; 95% CI: 0.76–0.97). An association was observed with cough (AOR: 1.09; 95% CI: 1.02–1.17) in East Africa only.

### 3.6. Role of Time Period Survey Was Undertaken

In the stratified sub-analysis of those surveys undertaken during or after 2015, an association within an increase in the adjusted odds ratio of fever (AOR: 1.18; 95% CI: 1.10–1.26), ARI (AOR: 1.11; 95% CI: 1.01–1.22), severe ARI (AOR: 1.17; 95% CI: 1.04–1.31) in children under five years old living in wood compared to charcoal cooking households (Table 2). However, no associations were observed in surveys undertaken during or before 2014.

## 4. Discussion

In our large cross-sectional multi-country study (30 countries; 475,089 participants), increased odds ratios of ARI were observed in Asia (AOR: 1.09; 95% CI: 1.05–1.13) and East Africa only (AOR: 1.04; 95% CI: 1.00–1.08) among children living in wood cooking households compared to charcoal cooking households. The risk of ARI varies between countries, and this may reflect different wood fuel choices, cultural differences, access to healthcare [49], elevation [50] and seasonal or climatic differences [51], which could not be accounted for in our analyses. Moreover, the variation of the observed outcome results between countries indicates the need to take current country and regional level characteristics into consideration when developing HAP interventions for reducing ARI in children aged under five years. This is further highlighted as only the most recent surveys (post-2015) have an observed association with fever (AOR: 1.18; 95% CI: 1.10–1.26), ARI (AOR: 1.11; 95% CI: 1.01–1.22), severe ARI (AOR: 1.17; 95% CI: 1.04–1.31). Prevention of ARI in children aged under five years would reduce child mortality and long-term morbidities, exert health and fiscal benefits; in addition to supporting progress towards the Sustainable Development Goals (SDGs) (namely SDG 3).

Given the wider environmental and health impacts of charcoal production and use, there is an argument for restricting the use of charcoal. However, the clear financial and structural difficulties of provision of clean fuels in many LMIC settings means charcoal restriction could potentially result in health harms by some users reverting to other biomass fuels. Although charcoal use presents significant health harms [52], it has previously been shown to produce lower pollutant levels than wood cooking in laboratory and field studies [15,18], and our results reflect these findings and their effect on child health, indicating that compared to charcoal, wood cooking is associated with increased risk of ARI in East Africa and Asia. It is evident that a package of measures is required for fuel transition policies which include charcoal restrictions, to limit increased uptake of wood alternatives. Adoption of charcoal restrictions should be carefully considered, in terms of the potential health harms, in LMIC settings, in the context of clean fuel access, availability and affordability. Given the volatility of fuel costs, for example in response to disruptive changes such as COVID-19, such policies must also consider the longer-term resilience of domestic fuel supplies, in each specific context.

Fuel choice, preparation, cooking characteristics, and cumulative exposure have been shown to vary between countries [53]. We also explored how cooking location (indoor vs outdoor) and exposure to second-hand smoke from household smoking could potentially contribute to the risk of ARI and severe ARI. In the main analysis outdoor cooking was associated with reduced risk of shortness of breath, cough, fever and ARI (AOR: 0.96; 95% CI: 0.94–0.98]), which is another potential interim harm mitigation behavioural intervention promoted to reduce the adverse health effects of HAP exposure [10]. However, in the sub-analysis of outdoor cooking and indoor cooking separately, an association was only observed with fever (AOR: 1.07; 95% CI: 1.00–2.13) in indoor cooking households, which may have resulted from the small sample size or influence of seasonal factors [51]. However, a more detailed country-specific assessment by differing solid biomass fuels [40] would be required to understand the overall potential benefits of cooking outdoors; in addition, to the combined health effects and pollutants level benefits when changing cooking fuel to charcoal and moving cooking outside.

Household smoking could not be accounted for in the main summary analysis, due to missing data in Peru, the Philippines, and Kenya DHS surveys. In the exploratory analysis, limited effect of household smoking was observed upon outcome measures. Smoking is not only an alternative source of HAP exposure but is also a recognised risk factor for respiratory infections in infants [54]. However, an association was only observed in households with a smoker with cough (AOR: 1.05; 95% CI: 1.01–1.08) and fever (AOR: 1.04; 95% CI: 1.01–1.07), compared to non-smoking households, indicating the limited potential of smoking causing the occurrence of ARI.

Urban and rural areas also have additional differing situational contexts, including housing type, co-inhabiting with livestock, food security, WASH, household crowding, malnutrition, access to healthcare, wealth [55] and ambient pollution levels [56]. Differences in changes over time were investigated through the sub-analysis comparing surveys between 2010–2014 (n = 15) and 2015–2018 (n = 15), which indicated potential differing situational contexts. The role of alternative sources of HAP and differing situational characteristics both within and between countries highlights the complexities that need to be considered to understand the context-specific needs and acceptability of behavioural harm reduction HAP interventions.

Although there is an environmental and health need for reducing the reliance on charcoal cooking fuel, legislative approaches to restricting charcoal use should take into consideration the potential unintended or unanticipated health consequences of targeted fiscal policies. Wood fuels are readily available in most settings, as they are typically free to collect, thus may be reverted to as a fuel of choice [57], along with being strongly linked with poverty [58]; as seen in this study with the wealth index. This combination of factors increases the vulnerability of households to the health-harms of solid biomass cooking. Other approaches to improving the sustainability of charcoal such as improving sustainable production and the use of ICS for improved burning efficiency [17,59,60], could be considered as alternative mitigation measures in the short to medium term. However, the longer-term solution is to support the sustained adoption of cleaner fuels, with maintained strong supply links [61] to prevent fuel switching, as seen in the COVID-19 lockdowns [23,27]; and also provide education for health-harms of using solid biomass cooking fuel. Any policy mitigation measures for HAP to reduce ARIs in children under five should also consider wider protective health behaviours against ARIs, e.g., encouraging breastfeeding, especially within the first 6 months of life [62,63], childhood vaccinations, undernutrition [4], reducing the incidence of HIV, TB [64], and reducing the risk of low birth weight [65].

Although the use of fuel type as a proxy for HAP exposure, self-reported respiratory symptoms, changes over time, weaknesses in the potential to control for all confounding factors and the observational nature of the data generate study limitations, this population-based approach provides a large sample size and global comparison, detailing the widespread impact of a potential harm reduction intervention for HAP exposure. In addition, many potential associations were investigated; therefore, some association would be expected to be down to chance. Further research implications include the need to characterise exposure levels and exposure-response functions for key health outcomes and increased clinical diagnostic confirmation to improve aetiological specificity. Further research is also necessary to understand the specific physiological mechanism between specific pollutant exposure and ARI risk in children aged under five years, including by specific wood and charcoal types and combustion techniques. Further, we recommend consideration of the implications of wood to charcoal transition for climate change, specifically for CO_2_ emissions [66] and environmental degradation associated with charcoal production [67]. This study has global implications and provides the evidence to support a clear policy recommendation for safer domestic cooking practices.

## 5. Conclusions

Our population-based observational study indicates that in Asia and East Africa there is a greater risk of ARI among children aged under 5 years living in wood compared to charcoal cooking households. Users of domestic wood fuels are among the most vulnerable sub-populations worldwide and our findings support the need for ensuring long-term uptake of clean domestic energy alternatives in resource-poor settings worldwide. Policymakers should adopt an evidence-based approach, to ensure long-term sustained uptake of clean domestic energy alternatives and to prevent unintended consequences of biomass fuel restriction policies.

## Figures and Tables

**Table 1:** Descriptive statistics before imputation for respiratory health outcomes (N = 475,089).

	Cough (*N* = 442,450, Missing = 6.9%)			Shortness of Breath (*N* = 442,040, Missing = 7.0%)			Fever (*N* = 442,550, Missing = 6.9%)			ARI (*N* = 441,987, Missing = 7.0%)			Severe ARI (*N* = 441,627, Missing = 7.0%)		
	No (N = 359,927) n (%)	Yes (N = 82,523) n (%)	*p* Value	No (N = 403,337) n (%)	Yes (N = 38,703) n (%)	*p* Value	No (N = 352,929) n (%)	Yes (N = 89,621) n (%)	*p* Value	No (N = 405,330) n (%)	Yes (N = 36,657) n (%)	*p* Value	No (N = 418,137) n (%)	Yes (N = 23,490) n (%)	*p* Value
**Household cooking fuel**			<0.001			<0.001			<0.001			<0.001			0.790
Charcoal	36,279 (10.1%)	12,296 (14.9%)		43,749 (10.8%)	4799 (12.4%)		37,859 (10.7%)	10,774 (12.0%)		44,033 (10.9%)	4500 (12.3%)		45,933 (11.0%)	2546 (10.8%)	
Wood	323,648 (89.9%)	70,227 (85.1%)		359,588 (89.2%)	33,904 (87.6%)		315,070 (89.3%)	78,847 (88.0%)		361,297 (89.1%)	32,156 (87.7%)		372,204 (89.0%)	20,944 (89.2%)	
**Child’s sex**			<0.001			0.064			<0.001			<0.001			<0.001
Male	183,227 (50.9%)	42,317 (51.3%)		205,018 (50.8%)	20,329 (52.5%)		179,258 (50.8%)	46,364 (51.7%)		206,066 (50.8%)	19,249 (52.5%)		212,678 (50.9%)	12,463 (53.1%)	
**Child’s Age (months)**			<0.001			<0.001			<0.001			<0.001			<0.001
0–11	76,440 (21.2%)	18,754 (22.7%)		85,537 (21.2%)	9575 (24.7%)		75,566 (21.4%)	19,644 (21.9%)		86,053 (21.2%)	9055 (24.7%)		89,380 (21.4%)	5693 (24.2%)	
12–23	69,761 (19.4%)	20,002 (24.2%)		80,043 (19.8%)	9614 (24.8%)		66,964 (19.0%)	22,806 (25.4%)		80,529 (19.9%)	9124 (24.9%)		83,344 (19.9%)	6261 (26.7%)	
24–35	69,555 (19.3%)	16,566 (20.1%)		78,494 (19.5%)	7556 (19.5%)		67,785 (19.2%)	18,370 (20.5%)		78,899 (19.5%)	7140 (19.5%)		81,311 (19.4%)	4671 (19.9%)	
36–47	72,650 (20.2%)	14,756 (17.9%)		80,785 (20.0%)	6552 (16.9%)		71,814 (20.3%)	15,601 (17.4%)		81,120 (20.0%)	6206 (16.9%)		83,422 (20.0%)	3785 (16.1%)	
48–59	71,521 (19.9%)	12,445 (15.1%)		78,479 (19.5%)	5405 (14.0%)		70,799 (20.1%)	13,200 (14.7%)		78,729 (19.4%)	5133 (14.0%)		80,680 (19.3%)	3080 (13.1%)	
**Birth order**			<0.001			<0.001			<0.001			<0.001			<0.001
Not first born	256,340 (71.2%)	60,619 (73.5%)		288,083 (71.4%)	28,569 (73.8%)		250,638 (71.0%)	66,433 (74.1%)		289,685 (71.5%)	26,928 (73.5%)		299,038 (71.5%)	17,343 (73.8%)	
Mode of delivery *			0.005			<0.001			0.099			<0.001			<0.001
Caesarean	25,107 (7.0%)	6134 (7.5%)		28,378 (7.1%)	2846 (7.4%)		25,064 (7.2%)	6182 (7.0%)		28,487 (7.1%)	2735 (7.5%)		29,437 (7.1%)	1777 (7.6%)	
Birthweight*			<0.001			<0.001			<0.001			<0.001			<0.001
Low	63,970 (28.8%)	11,814 (23.3%)		69,945 (28.0%)	5799 (25.3%)		62,841 (28.6%)	12,935 (24.4%)		70,163 (27.9%)	5578 (25.7%)		72,017 (27.8%)	3687 (26.5%)	
Breastfeeding status *			<0.001			<0.001			<0.001			<0.001			<0.001
Never Breastfed	14,047 (5.1%)	2197 (3.2%)		15,209 (4.9%)	1021 (3.2%)		13,850 (5.1%)	2402 (3.1%)		15,244 (4.8%)	981 (3.2%)		15,537 (4.8%)	674 (3.4%)	
**Vitamin A supplementation**			<0.001			<0.001			<0.001			<0.001			<0.001
Yes	193,115 (54.6%)	49,265 (60.9%)		219,310 (55.3%)	22,867 (60.2%)		190,098 (54.8%)	52,338 (59.4%)		220,415 (55.3%)	21,741 (60.5%)		227,872 (55.4%)	14,113 (61.3%)	
Iron supplementation *			0.106			<0.001			0.018			<0.001			<0.001
Yes	63,042 (18.4%)	12,534 (16.6%)		69,285 (18.1%)	6245 (17.5%)		60,992 (18.2%)	14,587 (17.3%)		69,500 (18.1%)	6026 (17.9%)		71,308 (18.0%)	4156 (19.1%)	
**Maternal age (years)**			0.012			<0.001			<0.001			<0.001			<0.001
15–24	121,452 (33.7%)	27,738 (33.6%)		135,727 (33.7%)	13,329 (34.4%)		119,212 (33.8%)	29,974 (33.4%)		136,313 (33.6%)	12,723 (34.7%)		140,647 (33.6%)	8244 (35.1%)	
25–35	189,953 (52.8%)	42,587 (51.6%)		212,443 (52.7%)	19,888 (51.4%)		186,522 (52.8%)	46,110 (51.5%)		213,507 (52.7%)	18,795 (51.3%)		220,127 (52.6%)	11,987 (51.0%)	
36–49	48,523 (13.5%)	12,198 (14.8%)		55,167 (13.7%)	5486 (14.2%)		47,195 (13.4%)	13,537 (15.1%)		55,509 (13.7%)	5138 (14.0%)		57,363 (13.7%)	3259 (13.9%)	
**Maternal education level**			<0.001			<0.001			<0.001			<0.001			<0.001
No education	143,818 (40.0%)	25,418 (30.8%)		155,811 (38.6%)	13,185 (34.1%)		136,969 (38.8%)	32,317 (36.1%)		156,599 (38.6%)	12,384 (33.8%)		160,619 (38.4%)	8196 (34.9%)	
Primary	98,665 (27.4%)	30,379 (36.8%)		114,886 (28.5%)	14,076 (36.4%)		98,262 (27.8%)	30,839 (34.4%)		115,753 (28.6%)	13,178 (36.0%)		120,526 (28.8%)	8302 (35.3%)	
Secondary/Higher	117,428 (32.6%)	26,724 (32.4%)		132,623 (32.9%)	11,442 (29.6%)		117,685 (33.3%)	26,461 (29.5%)		132,961 (32.8%)	11,095 (30.3%)		136,974 (32.8%)	6991 (29.8%)	
**Household wealth index**			<0.001			<0.001			<0.001			<0.001			<0.001
Lowest	100,447 (27.9%)	21,240 (25.7%)		110,928 (27.5%)	10,698 (27.6%)		97,093 (27.5%)	24,616 (27.5%)		111,451 (27.5%)	10,167 (27.7%)		114,771 (27.4%)	6773 (28.8%)	
Middle	80,643 (22.4%)	17,910 (21.7%)		90,194 (22.4%)	8240 (21.3%)		78,956 (22.4%)	19,595 (21.9%)		90,616 (22.4%)	7810 (21.3%)		93,332 (22.3%)	5000 (21.3%)	
Highest	27,563 (7.7%)	8875 (10.8%)		32963 (8.2%)	3434 (8.9%)		28,989 (8.2%)	7464 (8.3%)		33,149 (8.2%)	3237 (8.8%)		34,533 (8.3%)	1811 (7.7%)	
Household smoking *			<0.001			<0.001			<0.001			<0.001			<0.001
Yes	126,552 (36.2%)	24,092 (31.4%)		138,907 (35.6%)	11,612 (32.2%)		123,113 (36.1%)	27,526 (32.2%)		139,279 (35.5%)	11,231 (33.0%)		142,874 (35.4%)	7518 (34.3%)	
**Household cooking location**			0.118			0.395			<0.001			0.001			<0.001
Indoors	262,449 (73.2%)	60,621 (73.8%)		293,885 (73.1%)	28,871 (75.0%)		258,651 (73.5%)	64,479 (72.4%)		295,282 (73.1%)	27433 (75.3%)		304,800 (73.2%)	17,651 (75.6%)	
Number of household member *			<0.001			<0.001			<0.001			<0.001			<0.001
≤6	174,300 (48.5%)	44,496 (53.9%)		198,285 (49.2%)	20,349 (52.6%)		172,461 (48.9%)	46,326 (51.7%)		199,396 (49.2%)	19,201 (52.4%)		206,328 (49.4%)	12,071 (51.4%)	
**Place of residence**			0.476			<0.001			0.048			<0.001			0.578
Urban	66,652 (18.5%)	17,978 (21.8%)		77,206 (19.1%)	7349 (19.0%)		67,436 (19.1%)	17,232 (19.2%)		77,496 (19.1%)	7049 (19.2%)		80,271 (19.2%)	4201 (17.9%)	

*N* = observation number, n = category observation number, % = column percentage for category. *p* value = Chi-Squared. * Missing data = Mode of delivery = 0.7%, Breastfeeding status = 22.8%, Birthweight = 38.3%, Vitamin A supplementation = 1.7%, Iron Supplementation = 534%, Mother’s education = 0.004%, Household smoking = 3.6%, Cooking location = 0.4%, Number of household members = 0.06%.

**Table 2:** Summary effects (AOR–95% CI) for respiratory symptoms, ARI and severe ARI with wood cooking of the whole, exploratory and sub-analysis.

Analysis (N]	Cough AOR (95%CI)	Shortness of Breath AOR (95%CI)	Fever AOR (95%CI)	ARI AOR (95%CI)	Severe ARI AOR (95%CI)
Whole (N = 482,644)	0.99(0.95–1.04)	1.03(0.96–1.10)	**1.07(1.02–1.12) ^b^**	1.03(0.96–1.11)	1.07(0.99–1.17)
**Sub-analysis**					
Urban areas (N = 89,661)	0.93(0.87–1.00)	0.99(0.90–1.09)	1.03(0.96–1.10)	0.99(0.89–1.09)	1.02(0.90–1.14)
Rural Area (N = 392,983)	**1.08(1.01–1.15) ^c^**	1.00(0.90–1.11)	1.05(0.98–1.13)	1.02(0.92–1.14)	1.05(0.92–1.20)
Indoor (N = 368,647)	1.03(0.97–1.09)	1.05(0.96–1.14)	**1.07(1.00–2.13) ^c^**	1.06(0.97–1.15)	1.08(0.97–1.20)
Outdoor (N = 113,997)	0.96(0.89–1.04)	0.98(0.89–1.09)	1.07(0.99–1.16)	1.01(0.91–1.13)	1.10(0.96–1.25)
Africa (N = 245,363)	1.02(0.97–1.08)	1.00(0.93–1.08)	1.02(0.97–1.08)	1.01(0.93–1.09)	1.04(0.94–1.14)
Asia (N = 233,091)	1.05(0.92–1.20)	**1.25(1.04–1.51) ^b^**	1.06(0.93–1.20)	**1.25(1.04–1.51) ^c^**	1.24(0.99–1.54)
Central Africa (N = 47,710)	0.97(0.86–1.11)	0.99(0.79–1.24)	0.99(0.86–1.13)	0.99(0.79–1.25)	1.04(0.80–1.36)
East Africa (N = 105,543)	**1.09(1.02–1.17) ^b^**	**1.10(1.00–1.20) ^c^**	1.01(0.94–1.09)	**1.11(1.01–1.22) ^c^**	1.10(0.98–1.24)
West Africa (N = 92,110)	0.96(0.87–1.06)	**0.86(0.76–0.97) ^b^**	1.00(0.92–1.09)	0.88(0.78–1.00)	0.93(0.80–1.09)
Surveys undertaken during or before 2014 (N = 134,225)	0.97(0.91–1.04)	0.99(0.89–1.09)	1.01(0.94–1.08)	0.99(0.89–1.09)	1.00(0.89–1.14)
Surveys undertaken during or after 2015 * (N = 348,419)	1.04(0.97–1.12)	1.09(1.00–1.20)	**1.18(1.10–1.26) ^a^**	**1.11(1.01–1.22) ^c^**	**1.17(1.04–1.31) ^b^**

AOR = adjusted odds ratio for wood cooking compared to charcoal, 95% CI = 95% confidence interval, a = *p* ≤ 0.001, b = *p* ≤ 0.01, c = *p* ≤ 0.05, bold = *p* ≤ 0.05, N = number of observations in the SARI analysis. * Surveys that were undertaken across 2014–2015 (n = 2) were included within during or after 2015 (Total countries = 15).

## Data Availability

Data is freely and publicly available at: https://dhsprogram.com/ (accessed on 15 January 2020).

## Appendix B

**Table A1:** Predictors included in the principal component analysis for the calculation of the modified wealth index within each country.

Country	Source of Drinking Water	Toilet Facility	House Construction			Assets																			Dwelling Window Material	Lighting Fuel	Refuse Collection	Own or Rent House
			Wall Material	Roof Material	Floor Material	Electricity	Radio	Television	Refrigerator	Watch	Bicycle	Motorcycle or Scooter	Animal-Drawn Cart	Car or Truck	Boat with a Motor	Bank Account	Mobile Telephone	Computer	Non-Motorised Boat or Canoe	Tractor	Plough	Household Furniture *	Household Electronics *	Other Assets *				
Afghanistan 2015																												
Benin 2017–2018																												
Burkina Faso 2010																												
Burundi 2016–2017																												
Cambodia 2014																												
Cameroon 2018																												
Chad 2014–2015																												
Congo 2011–2012																												
Côte d'Ivoire 2011–2012																												
DRC 2014–2015																												
Ethiopia 2016																												
Gambia 2013																												
Ghana 2014																												
Guinea 2018																												
India 2015–2016																												
Indonesia 2012																												
Kenya 2014																												
Liberia 2013																												
Malawi 2015–2016																												
Mozambique 2011																												
Myanmar 2015–2016																												
Nigeria 2018																												
Pakistan 2013																												
Peru 2012																												
Philippines 2013																												
																												
Rwanda 2014–2015																												
Sierra Leone 2013																												
Tanzania 2015–2016																												
Uganda 2016																												
Zambia 2013–2014																												

* Household furniture (e.g., table, chairs, wardrobe, bed, mattress, lamps, clock). * Household electronics (e.g., washing machine, DVD player, internet, modem/router, satellite, laptop, generator, music system, sewing machine, fan, air conditioning, solar panel, water pump, battery, iron, TV5 antenna, Cable subscription, camera, blender, microwave). * Other assets (e.g., Grain mill, hammer mill, Rickshaw/chingchi/Tuk tuk/htawlargyi/Keke Napep/Bagag, bank account with another institution, credit union, beneficiary of Pantawid Pamilyan Pilipino Program (4Ps), canoe with motor, banana boat, thresher, bedroom available for sleep, floor area of house).

## Appendix C

**Table A2:** Summary effects (AOR–95% CI) for respiratory symptoms, ARI and severe ARI with wood cooking for the exploratory analysis.

Analysis (N)	Cough AOR (95%CI)	Shortness of Breath AOR (95%CI)	Fever AOR (95%CI)	ARI AOR (95%CI)	Server ARI AOR (95%CI)
Controlling for birthweight ^1^ (N = 405,839)	0.97(0.93–1.03)	1.02(0.94–1.10)	1.08(1.02–1.14) ^a^	1.02(0.94–1.10)	1.02(0.94–1.10)
Controlling for breastfeeding ^2^ (N = 448,769)	0.97(0.92–1.02)	1.01(0.94–1.09)	1.07(1.01–1.12) ^b^	1.02(0.94–1.10)	1.07(0.98–1.18)
Controlling for household smoking ^3^ (N = 455,289)	0.98(0.93–1.03)	1.01(0.94–1.09)	1.06(1.01–1.12) ^c^	1.02(0.94–1.10)	1.07(0.97–1.17)

AOR = adjusted odds ratio for wood cooking compared to charcoal, 95% CI = 95% confidence interval, a = *p* ≤0.001, b = *p* ≤0.01, c = *p* ≤0.05, bold = N = *p* ≤0.05, number of observations in the SARI analysis. ^1^ = Chad, Ethiopia, Kenya, Guinea, Liberia, Afghanistan, Myanmar, Pakistan, Nigeria were excluded due to < 50% missing data for birthweight. ^2^ = Peru, Zambia and Kenya were excluded due to < 50% missing data for breastfeeding. ^3^ = Household smoking data not collected for Kenya, Peru and the Philippines.
